# T1 Bladder Cancer: Comparison of the Prognostic Impact of Two Substaging Systems on Disease Recurrence and Progression and Suggestion of a Novel Nomogram

**DOI:** 10.3389/fsurg.2021.704902

**Published:** 2021-08-23

**Authors:** Anastasios D. Asimakopoulos, Gaia Colalillo, Rossana Telesca, Alessandro Mauriello, Roberto Miano, Savino Mauro Di Stasi, Stefano Germani, Enrico Finazzi Agrò, Vincenzo Petrozza, Gianluca Caruso, Antonio Carbone, Antonio Luigi Pastore, Andrea Fuschi

**Affiliations:** ^1^Division of Urology, Fondazione PTV Policlinico Tor Vergata, Rome, Italy; ^2^Division of Urology, Department of Surgical Sciences, University of Rome Tor Vergata, Rome, Italy; ^3^Pathology, Department of Experimental Medicine and Surgery, University of Rome Tor Vergata, Rome, Italy; ^4^Pathology, Department of Medical-Surgical Sciences and Biotechnologies, Sapienza University of Rome, Latina, Italy; ^5^Urology Unit ICOT, Department of Medico-Surgical Sciences and Biotechnologies, Faculty of Pharmacy and Medicine, Sapienza University of Rome, Latina, Italy

**Keywords:** bladder cancer, urothelial carcinoma, staging, lamina propria, prognosis, urinary bladder neoplasms

## Abstract

**Background:** The T1 substaging of bladder cancer (BCa) potentially impacts disease progression. The objective of the study was to compare the prognostic accuracy of two substaging systems on the recurrence and progression of primary pathologic T1 (pT1) BCa and to test a nomogram based on pT1 substaging for predicting recurrence-free survival (RFS) and progression-free survival (PFS).

**Methods:** The medical records of 204 patients affected by pT1 BCa were retrospectively reviewed. Substaging was defined according to the depth of lamina propria invasion in T1_a−*c*_ and the extension of the lamina propria invasion to T1-microinvasive (T1_m_) or T1-extensive (T1_e_). Uni- and multivariable Cox regression models evaluated the independent variables correlated with recurrence and progression. The predictive accuracies of the two substaging systems were compared by Harrell's C index. Multivariate Cox regression models for the RFS and PFS were also depicted by a nomogram.

**Results:** The 5-year RFS was 47.5% with a significant difference between T1_c_ and T1_a_ (*p* = 0.02) and between T1_e_ and T1_m_ (*p* < 0.001). The 5-year PFS was 75.9% with a significant difference between T1_c_ and T1_a_ (*p* = 0.011) and between T1_e_ and T1_m_ (*p* < 0.001). Model T1_m−*e*_ showed a higher predictive power than T1_a−*c*_ for predicting RFS and PFS. In the univariate and multivariate model subcategory T1e, the diameter, location, and number of tumors were confirmed as factors influencing recurrence and progression after adjusting for the other variables. The nomogram incorporating the T1_m−e_ model showed a satisfactory agreement between model predictions at 5 years and actual observations.

**Conclusions:** Substaging is significantly associated with RFS and PFS for patients affected by T1 BCa and should be included in innovative prognostic nomograms.

## Introduction

Up to 80% of bladder cancers (BCa) are non–muscle invasive during the first diagnosis (1); ~25% present as T1 lesions with invasion of the subepithelial connective tissue (i.e., lamina propria) (1–3).

The management of T1 BCa is controversial since it presents different degrees of aggressiveness, with a progression rate varying from 12 to 54% (4, 5). Many clinical (age, gender, multifocality, and tumor size) and pathologic (concomitant CIS, tumor grade, and architecture) factors for recurrence and progression have been extensively studied over the years to predict prognosis and guide management decisions (1, 6).

Younes et al. (7) were the first to use the muscularis mucosa (MM) in transurethrally resected biopsy specimens to substage (i.e., subcategorize) T1 BCa. Since then, several studies have been conducted to identify whether the depth of lamina propria invasion is a valuable prognostic factor with the use of the MM as a landmark for T1 substaging. This was done according to the location of the invasion: above the muscularis mucosa–vascular plexus (MM-VP) (T1_a_), in the MM-VP (T1_b_), or beyond the MM-VP (T1_c_). A recent meta-analysis demonstrated a worse prognosis for T1 tumors with deep MM invasion (8). However, other studies underlined that the substaging of T1 BCa is technically difficult because MM and VP represent inconsistent histologic landmarks for staging (i.e., not always present). Furthermore, if present, these structures can be overrun by the invasive tumor, making substaging challenging. In fact, a lack of consensus among pathologists regarding the identification of the MM-VP at the invasion front of the tumor has been reported, while others suggest that the T1 substaging does not add prognostic value with respect to survival (9, 10).

In 2009, van Rhijn (1) evaluated another, more “user-friendly” method intended as a more feasible substaging system that does not require the identification of the MM-VP to subcategorize T1 BCa and discern between T1-microinvasive (T1_m_) and T1-extensive-invasive (T1_e_) tumors, as described by van der Aa et al. (11). In their multivariable analyses, substage (T1_m−e_) was significant for progression and disease specific survival, whereas substage according to T1_a−c_ was not significant.

The objective of the study was to evaluate and compare the prognostic significance of two substaging systems (pT1_a−*c*_ and pT1_m−*e*_) on the rate of recurrence and progression of the T1 BCa. A novel nomogram that incorporates T1 substaging and depicts the probability of recurrence and progression-free survival was also created.

## Materials and Methods

Between 2009 and 2017, 787 patients underwent a transurethral resection (TUR) for BCa at two institutions; 240 of them were diagnosed as having primary pathologic T1 (pT1) urothelial BCa. Out of 240 patients, 30 were excluded for incomplete data while six were excluded because of concomitant urothelial carcinoma in the upper urinary tract. Consequently, the medical records of 204 patients were retrospectively reviewed.

Random biopsies, a standard repeat transurethral resection (re-TUR), and a single instillation of chemotherapy after TUR were not routinely performed. The surveillance of the patients consisted of cystoscopy and cytology every 3–4 months in the first 2 years and subsequently at a lower frequency (6–12 months) if no recurrence was detected. Upper urinary tract imaging was done every 1–2 years or when indicated by clinical suspicion.

Available data regarding gender, age, smoking, neutrophil to lymphocyte ratio, bladder neck or trigonal location of the tumor, tumor diameter < or >3 cm, number of tumors (1 vs. 2–7 vs. >8), grade, concomitant carcinoma *in situ* (Cis), presence of muscularis propria, lymphovascular invasion (LVI), tumor pattern (papillary vs. solid), presence of associated squamous metaplasia, performance of re-TUR, Bacillus Calmette-Guerin (BCG) instillations, recurrence and time to recurrence, progression and time to progression, performance of radical cystectomy, cancer-specific mortality, death for other causes, and follow-up calculated in months until the last clinical office visit or death was inserted in a customized, institutional review board-approved database (PTV registration number 255.19). Pathology information was recorded from the pathology report but was fully reevaluated as part of the study.

Recurrence was defined as the histological detection of BCa through transurethral bladder resection (TUR) or bladder biopsy following the first TUR or the re-TUR. Progression was defined as the development of muscle-invasive disease or distant metastasis.

The TUR specimens of each institution were reviewed by their respective dedicated uropathologists. Tumors were staged according to the recently published American Joint Committee on Cancer (AJCC) Staging Manual 8th edition (12). The World Health Organization (WHO) 1973 classification system for grade was used to determine a grade, since Pellucchi et al. demonstrated that this grading system better stratifies patients with lamina propria invasion (13). Thus, T1 substaging was defined according to the depth of lamina propria invasion using the Younes et al. (7) classification as follows: T1_a_, invasion of the lamina propria superficial to the level of the MM; T1_b_, invasion to the level of the MM; T1_c_, invasion through the level of the MM but superficial to the muscularis propria. If the MM-VP was not present at the invasion front, the case was assigned to T1_a_ or T1_c_ based on the extent of invasion into the lamina propria by looking at the MM-VP in tumor-free areas in the same or other TUR chips. Otherwise, if the associated vascular plexus could be identified, then it served as a marker for the MM level.

The definition of T1_m_ was a single focus of lamina propria invasion with a diameter of <0.5 mm (within one high-power field, objective × 40). Specimens showing a larger area with invasion or multiple microinvasive areas were considered T1_e_ (1).

Substages T1_a−*c*_ and T1_m−*e*_ were then inserted into the customized database.

### Statistical Analysis

Categorical variables were described in terms of frequency (*n*) and percentage (%). Continuous variables were described as mean and SD or, if not normally distributed, as median and interquartile range (from the 25th to 75th percentile).

The association between categorical variables was tested by chi-square or, when appropriate, by Fisher's exact test. Differences in continuous variables among more than two groups were tested with the Kruskal–Wallis test. Post-comparisons were performed applying the Mann–Whitney test.

A log-rank test was applied to compare the survival curves among the substages. Uni- and multivariable Cox regression models were performed to evaluate the independent variables that influence recurrence-free survival (RFS). Variables with a *p* < 0.1 at the univariable analysis were considered in the multivariable model. Due to the low number of events in the model on progression-free survival (PFS), a stepwise selection method was applied to select the best set of predictors.

To compare the prognostic performance of the two substaging systems (T1_a−*c*_-Model 1 vs. T1_m−*e*_-Model 2), a univariable cox regression model was performed; then the prediction accuracy of these two models was compared by comparing the Harrell's C indexes while Lincom Stata command was applied to test the difference between Harrell's C.

The multivariate Cox regression model for RFS and PFS was depicted by a nomogram, considering the probability of survival at 5 years. In the nomogram, we assigned a score from 0 to 100 corresponding to the value of each predictor. By adding the singular scores, a total score was obtained that corresponded to the 5-year survival probability.

The nomogram was subjected to bootstrap resamples for the reduction of overfit bias of predicted vs. observed values depicted in the calibration plot. For the Cox models, the predicted value was obtained at a 5-year time-point while the observed value was the corresponding Kaplan–Meier survival estimate.

Statistical analysis was performed using the STATA (version 16.1) and R software (version 3.3.4, Regression Modeling Strategies package. The R Foundation for Statistical Computing, Vienna, Austria).

In multiple comparisons, the Benjamini–Hochberg method was applied to adjust the *p* values. A *p* < 0.05 was considered statistically significant.

## Results

The demographic and clinicopathological data were retrospectively collected for 204 patients (Table 1).

**Table 1:** Clinical and demographic characteristics.

Patients		N	204
Age, years		mean (SD)	72 (9.5)
Sex	M	n (%)	180 (87%)
Smoking	Yes	n (%)	85 (41.7%)
Neutrophil/lymphocyte, *n* = 191		Median (25–75 percentile)	4 (2.37–6.36)
Trigone/bladder neck	Yes	*n* (%)	65 (30.9%)
Tumor diameter, *N* = 203	>3 cm	*n* (%)	43 (21.2%)
N of tumors	1	*n* (%)	106 (52%)
	2–7	*n* (%)	88 (43.1%)
	≥8	*n* (%)	10 (4.9%)
Substage T1a-c	T1a	*n* (%)	97 (47.6%)
	T1b	*n* (%)	49 (24%)
	T1c	*n* (%)	39 (19.1%)
	Not evaluable (NV)	*n* (%)	19 (9.3%)
Substage T1m-e	T1m	*n* (%)	90 (44.1%)
	T1e	*n* (%)	79 (38.7%)
	Not evaluable (NV)	*n* (%)	35 (17.2%)
Grade	G1	*n* (%)	28 (13.7%)
	G2	*n* (%)	1 (0.5%)
	G3	*n* (%)	175 (85.8%)
CIS	Yes	*n* (%)	5 (2.5%)
Muscolaris propria	Yes	*n* (%)	167 (81.9%)
LVI, *n* = 203	Yes	*n* (%)	24 (11.8%)
Tumor pattern	Solid	*n* (%)	2 (1%)
Squamous metaplasia	Yes	n (%)	8 (3.9%)
Re-turb within 4 weeks, *n* = 203	Yes	*n* (%)	41 (20.2%)
BCG instillations, *n* = 202	Yes	*n* (%)	140 (70.4%)
Follow up, months		Median (25–75 percentile)	37.5 (21 −51.5)

*N, number; SD, standard deviation; M, male; CIS, carcinoma in situ; LVI, lymphovascular invasion; TURB, transurethral resection of bladder; BCG, bacillus Calmette-Guérin*.

*Not evaluable (NV) =T1 cases of bladder cancer where substaging was not feasible for several reasons such as*.

*-artifacts of the microscope slides*.

*-absence of the muscularis mucosae or its thinness, fragmentation and/or coagulation related to TUR*.

*-practical difficulties in defining the level of tumor invasion according to the orientation of specimens*.

With a median follow-up of 37.5 months (IQR 21–51.5), 106 patients experienced a recurrence, and 45 showed progression of the disease (Table 2). The association between substages T1_a−*c*_ and other clinical and demographic characteristics is shown in Table 3, while the association between substages T1_m−*e*_ and other variables is shown in Table 4.

**Table 2:** Recurrence, progression and mortality in the current series.

Patients	N	204
Recurrence	*n* (%)	106 (52%)
Progression	*n* (%)	45 (22.1%)
Radical cystectomy, *n* = 202	*n* (%)	35 (17.3%)
Cancer specific mortality, *n* = 165	*n* (%)	14 (8.5%)
Death for other cause, *n* = 156	*n* (%)	13 (8.3%)

**Table 3:** Association between Subcategory T1a-c and other characteristics.

		**Substage**					
		**T1a**	**T1b**	**T1c**	**NV**	***p***	**B-H adjusted *p***
		***n* = 97**	***n* = 49**	***n* = 39**	***n* = 19**		
**Smoke**						**0.001**	**0.002**
Yes	*n* (%)	36 (37.1%)	14 (28.6%)	27 (69.2%)	8 (42.1%)		
**Neutrophil/lymphocyte**							
	Median (25–75 percentile)	3.8 (2.11–5.8)	4.34 (2.59–6.3)	5.8 (3.7–7.6)	1.65 (1.28–2.86)	**0.0001** ^**a**^	**0.001**
**Trigone/bladder neck**						**0.001**	**0.002**
Yes	*n* (%)	30 (30.9%)	6 (12.2%)	20 (51.3%)	7 (36.8%)		
**Tumor diameter**, ***N*****= 203**						**<0.001**	**0.002**
>3 cm	*n* (%)	16 (16.7%)	6 (12.2%)	18 (46.2%)	3 (15.8%)		
***N*** **of tumors**						0.594	0.594
1	*n* (%)	46 (47.4%)	29 (59.2%)	18 (46.2%)	13 (68.4%)		
2–7	*n* (%)	46 (47.4%)	18 (36.7%)	19 (48.7%)	5 (26.3%)		
≥8	*n* (%)	5 (5.2%)	2 (4.1%)	2 (5.1%)	1 (5.3%)		
**Grade**						0.003^*^	**0.006**
G1	*n* (%)	21 (21.7%)	4 (8.2%)	0 (0%)	3 (15.8%)		
G2	*n* (%)	1 (1%)	0 (0%)	0 (0%)	0 (0%)		
G3	*n* (%)	75 (77.3%)	45 (91.8%)	39 (100%)	16 (84.2%)		
**Cis concomintant**						0.02^*^	**0.031**
Yes	*n* (%)	1 (1%)	0 (0%)	4 (10.3%)	0 (0%)		
**Muscolaris propria**						**0.019**	**0.031**
Yes	*n* (%)	76 (78.4%)	38 (77.6%)	38 (97.4%)	15 (79%)		
**Lymphovascular invasion**						**<0.001**	**0.001**
Yes	*n* (%)	4 (4.2%)	5 (10.2%)	14 (35.9%)	1 (5.3%)		
**Tumor pattern**						0.363	0.391
Papillary	*n* (%)	97 (100%)	48 (98%)	38 (97.4%)	19 (100%)		
**Squamous metaplasia**, ***N*****= 203**						0.323	0.377
Yes	*n* (%)	5 (5.2%)	0 (0%)	2 (5.1%)	1 (5.3%)		
**Re-turb**, ***N*****= 203**						0.055	0.077
Yes	*n* (%)	19 (19.8%)	12 (24.5%)	3 (7.7%)	7 (36.8%)		
**BCG**, ***N*****= 199**						0.073	0.093
Yes	*n* (%)	64 (68.1%)	30 (63.8%)	34 (87.2%)	12 (63.2%)		
**Radical cystectomy**, ***N*****= 202**						**<0.001**	**0.001**
Yes	*n* (%)	10 (10.3%)	7 (14.6%)	16 (41%)	2 (11.1%)		

*NV, not evaluable; B-H, Benjamini-Hockberg*.

* *Fisher exact test*.

a *Kruskal Wallis test. Post hoc comparisons showed: Non significant difference between T1a vs T1b (p = 0.314); Significant difference between T1a vs T1c (p = 0.006), vs NV (p = 0.007); Non significant difference between T1b vs T1c (p = 0.056), significant difference between T1b vs NV (p = 0.006); Significant difference between T1c vs NV (p = 0.001). All p values were adjusted for multiple comparisons (Benjamini-Hockberg method)*.

*Bold indicate the p value < 0.05*.

**Table 4:** Association between subcategory T1m-e and other characteristics.

		**Substage T1m-e**				
		**T1m**	**T1e**	**NV**	***p***	**B-H adjusted *p***
		***n* = 90**	***n* = 79**	***n* = 35**		
**Smoke**					0.766	0.766
**Yes**	*n* (%)	35 (38.9%)	35 (44.3%)	15 (42.9%)		
**Neutrophil/lymphocyte**						
	Median (25-75 percentile)	4(2.76 – 6.36)	3.7 (2.02 – 6)	4.75(1.64 – 7.3)	0.262^a^	0.435
**Trigone/bladder neck**					0.11	0.257
Yes	*n* (%)	22 (24.4%)	31 (39.2%)	10 (28.6%)		
**Tumor diameter**, ***N =*****203**					**<0.001**	**0.001**
>3 cm	*n* (%)	11 (12.4%)	28 (35.4%)	4 (11.4%)		
***N*** **of tumors**					0.24	0.435
1	*n* (%)	49 (54.4%)	34 (43%)	23 (65.7%)		
2-7	*n* (%)	37 (41.1%)	40 (50.6%)	11 (31.4%)		
≥8	*n* (%)	4 (4.4%)	5 (6.3%)	1 (2.9%)		
**Grade**					0.040^*^	0.112
G1	*n* (%)	18 (20%)	5 (6.3%)	5 (14.3%)		
G2	*n* (%)	1 (1.1%)	0 (0%)	0 (0%)		
G3	*n* (%)	71 (78.9%)	74 (93.7%)	30 (85.7%)		
**Cis concomintant**					0.019^*^	0.067
Yes	*n* (%)	0 (0%)	5 (6.3%)	0 (0%)		
**Muscolaris propria**					0.432	0.550
Yes	*n* (%)	74 (82.2%)	62 (78.5%)	31 (88.6%)		
**Lymphovascular invasion**					**<0.001**	**0.001**
Yes	*n* (%)	4 (4.4%)	18 (23.1%)	2 (5.7%)		
**Tumor pattern**					0.311^*^	0.435
Papillary	*n* (%)	90 (100%)	77 (97.5%)	35 (100%)		
**Squamous metaplasia**, ***N*****= 203**						
Yes	*n* (%)	5 (5.6%)	2 (2.5%)	1 (2.9%)	0.71	0.765
**Re-turb, N = 203**						
Yes	*n* (%)	15 (16.9%)	18 (22.8%)	8 (22.9%)	0.577	0.673
**BCG instillations**, ***N*****= 199**						
Yes	*n* (%)	57 (64.8%)	57 (75%)	26 (74.3%)	0.307	0.435
**Radical cystectomy**, ***N*****= 202**						
Yes	*n* (%)	8 (8.9%)	25 (32.1%)	2 (5.9%)	**<0.001**	**0.001**

*NV, not evaluable*.

* *Fisher exact test*.

a *Kruskal Wallis test*.

*B-H, Benjamini-Hockberg method to adjust p values for multiple comparisons*.

*Bold indicate the p value < 0.05*.

Among the subcategories T1_a−*c*_, T1_c_ had a significantly higher rate of both recurrence (71.8%) and progression (38.5%). Among the subcategories T1_m−*e*_, T1_e_ had a significantly higher rate of recurrence (70.9%) and progression (39.2%) (Table 5).

**Table 5:** Recurrence and progression by subcategory.

	**Substage T1a-c**				
	**T1a**	**T1b**	**T1c**	**NV**	
Recurrence	45 (46.39%)	25 (51.02%)	28 (71.79%)	8 (42.11%)	0.044
Progression	16 (16.49%)	11 (22.45%)	15 (38.46%)	3 (15.79%)	0.040
	**Substage T1m-e**				
	**T1m**	**T1e**		**NV**	
Recurrence	40 (44.44%)	56 (70.89%)		10 (28.57%)	<0.001
Progression	11 (12.22%)	31 (39.24%)		3 (8.57%)	<0.001

### Recurrence-Free Survival

**Figure 1:**
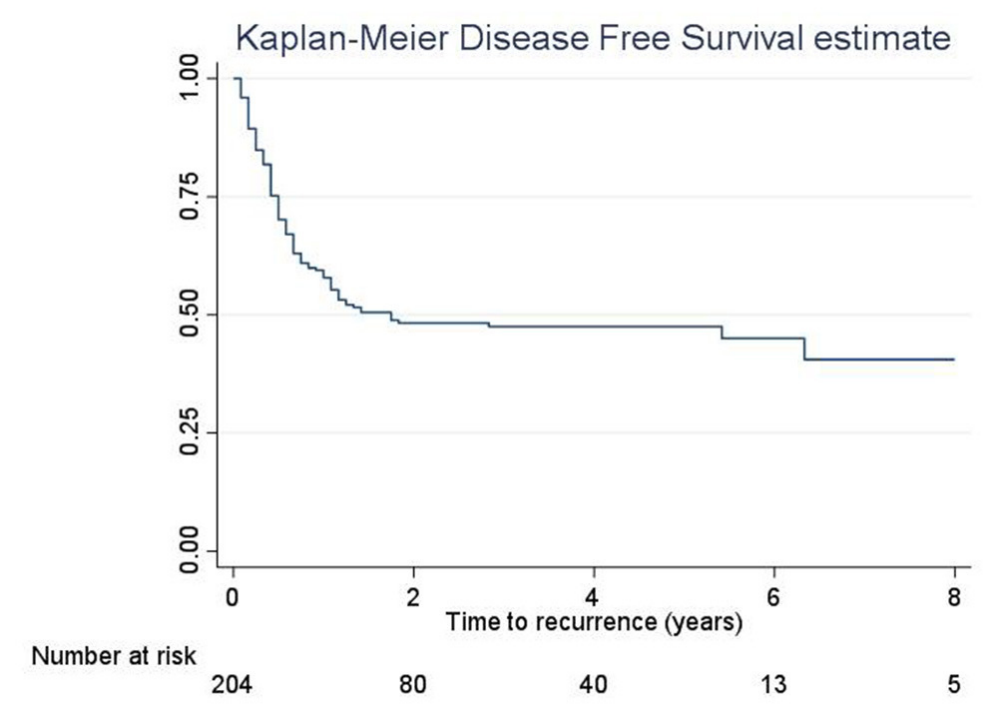
Recurrence-free survival, overall.

**Figure 2:**
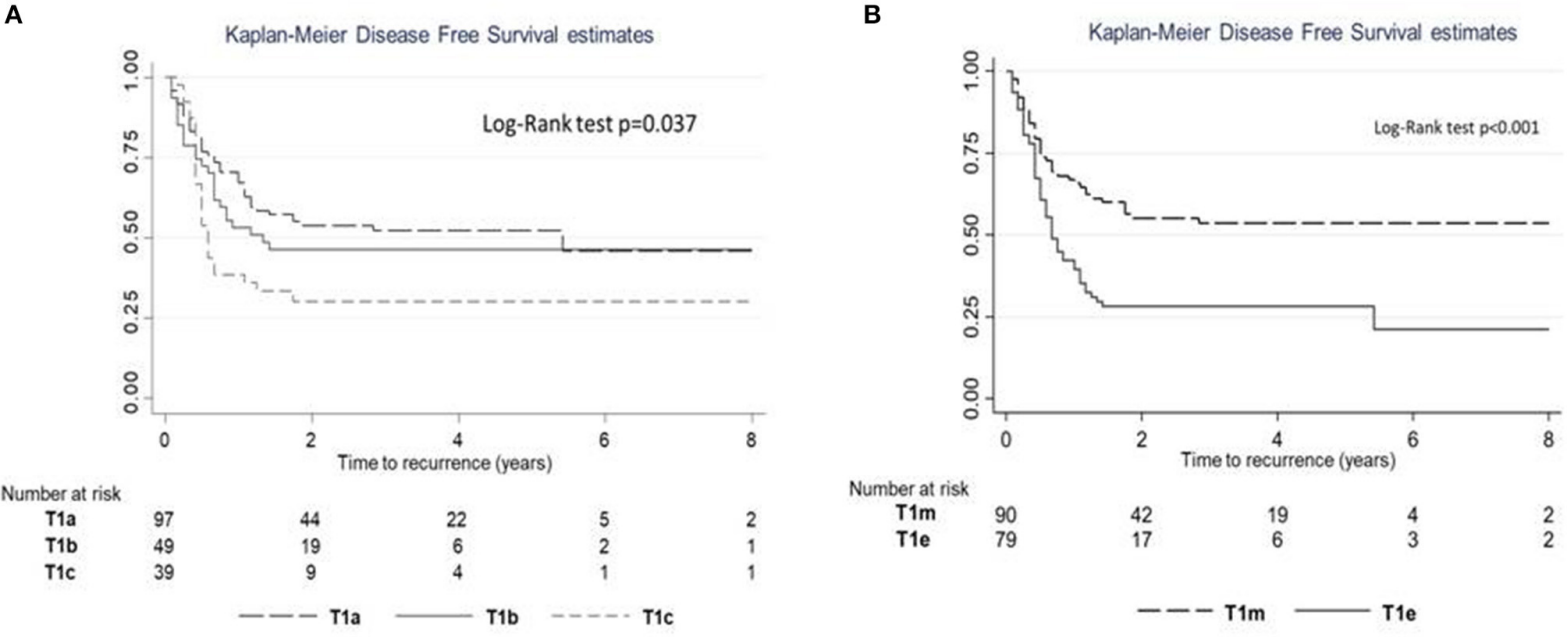
Recurrence-free survival (RFS) estimates according to subcategory. **(A)** RFS for subcategories T1a–c. **(B)** RFS for subcategories T1m–e.

The 5-year RFS was 47.5% (SE = 3.6%, Figure 1). The mean and median RFS was about 3.99 and 1.75 years, respectively. The RFS per substage is shown in Figure 2.

Harrell's C of model 1 (M1) was equal to 0.563, while Harrell's C of model 2 (M2) was 0.616. Comparing the predictive power, the M2 model seemed to have a slightly higher predictive power (difference = 0.053; *p* = 0.033).

**Figure 3:**
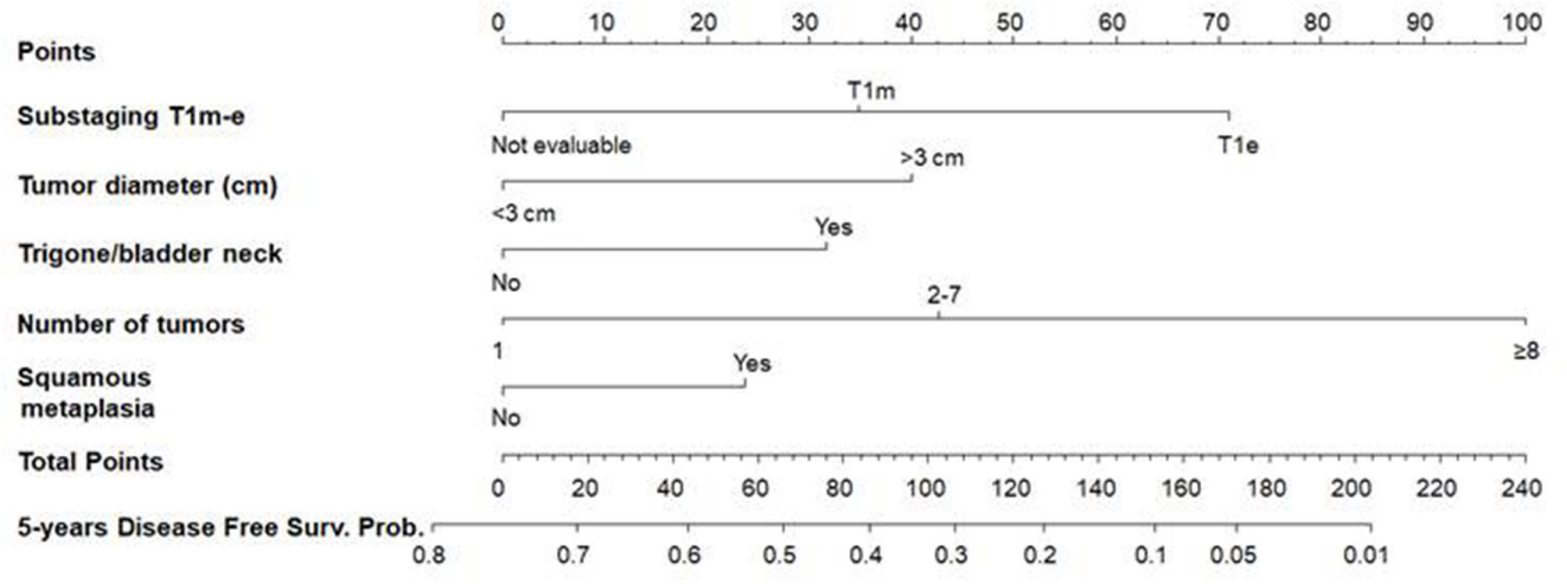
Nomogram 1 for the calculation of the recurrence-free survival (RFS) at 5 years. On the basis of the estimated Cox regression model, patients with T1e (71 points), a >3 cm tumor diameter (40 points), trigone or bladder neck location (32 points), with a number of tumors ≥8 (100 points), and presence of squamous metaplasia (24) had a total score of 267, which corresponds to a low 5-year RFS probability (*p* < 0.01).

**Figure 4:**
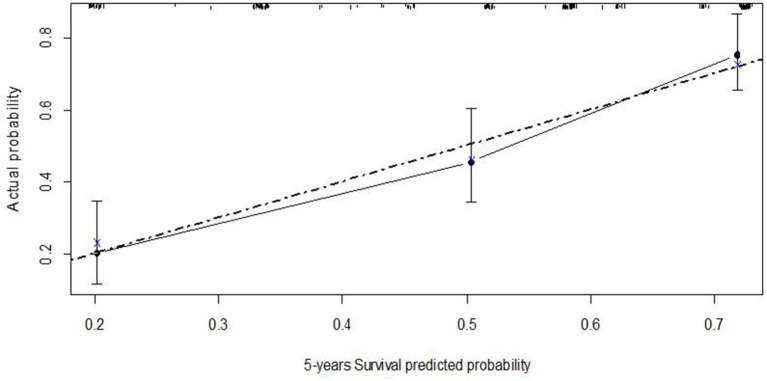
Calibration plot of the Cox regression model of recurrence-free survival at five years–nomogram 1.

Uni- and multivariable Cox regression models were performed to evaluate which independent variables influence recurrence (Table 6). Substages T1_a−*c*_ and T1_m−*e*_ were highly associated; however, since, in the comparison between models, M2 showed a higher predictive power, it was the only one considered in the multivariable analysis. This analysis was depicted by nomogram 1 (Figure 3). The calibration plot was depicted in Figure 4, showing a satisfactory agreement between model predictions at 5 years and actual observations.

**Table 6:** Uni and multivariable Cox regression model was performed to evaluate which independent variables influence the recurrence.

		**Univariable**				Multivariable^*^			
			**95% CI**				**95% CI**		
		**HR**	**LL**	**UL**	**p**	**HR**	**LL**	**UL**	**p**
**Age, yrs**		1	0.98	1.02	0.95				
**Sex**	M vs. F	0.95	0.53	1.7	0.873				
**Smoking**		1.1	0.75	1.63	0.616				
Neutrophil/lymphocyte, *n* = 191		0.93	0.85	1.01	**0.074**				
Trigone/bladder neck		1.71	1.15	2.54	**0.008**	1.5	0.99	2.27	0.055
Tumor diameter, *N*=203									
	>3 cm vs. <3 cm	1.93	1.25	2.98	**0.003**	1.7	1.05	2.75	0.032
**N of tumors**									
	2-7 vs. 1	1.7	1.13	2.55	**0.01**	1.75	1.13	2.7	0.012
	≥8 vs. 1	4.84	2.33	10.06	**<0.001**	3.68	1.66	8.19	0.001
**Substage T1a-c**									
	T1b vs. T1a	1.24	0.76	2.02	0.393	Not in the model			
	T1c vs. T1a	1.8	1.12	2.91	0.016				
	Not evaluable vs. T1a	0.9	0.41	2.01	0.802				
**Substage T1m-e**									
	T1e vs. T1m	2.03	1.35	3.07	0.001	1.59	1.02	2.46	0.039
	Not evaluable vs. T1m	0.544	0.26	1.12	0.1	0.57	0.28	1.19	0.136
**Grade**									
	G3 vs. G1	1.03	0.59	1.81	0.921				
**CIS**		1.93	0.71	5.26	0.2				
Muscolaris propria present		0.73	0.45	1.17	0.19				
Lymphovascular invasion, *n* = 203		1.62	0.96	2.74	**0.069**				
Tumor pattern	Solid	1.24	0.17	8.91	0.831				
Squamous metaplasia	Yes vs. no	2.31	1.01	5.29	**0.048**	1.39	0.55	3.51	0.482
BCG instillations *n* = 199	Yes	1.4	0.87	2.25	0.162				

*Bold indicate the p value < 0.10*.

* *N = 203*.

*In the final model the following variables were excluded: LVI because it was highly correlated with Substaging T1m-e and Neutrophil/lymphocyte in order to avoid a significant reduction of the sample size*.

### Progression-Free Survival

**Figure 5:**
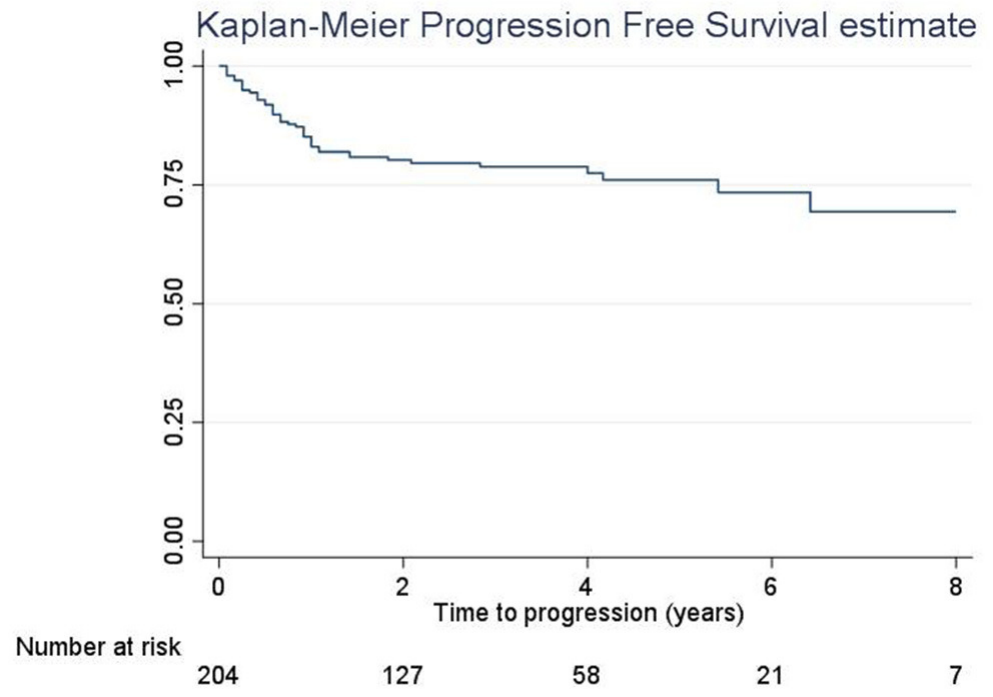
Progression-free survival, overall.

The 5-year PFS was 75.9% (SE = 3.5%, Figure 5). The PFS median time was not evaluable because the survival curve did not reach 50%; in fact, the overall PFS was higher than 50%. Overall mean PFS time was 6.2 years.

**Figure 6:**
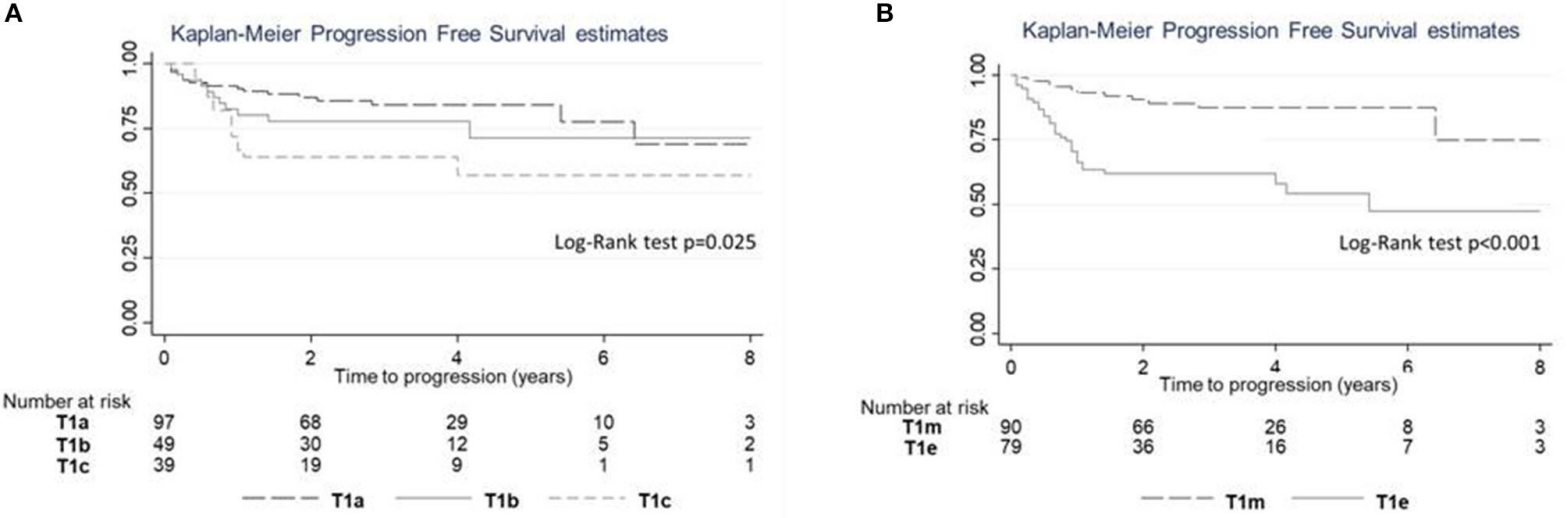
Progression-free survival (PFS) estimates according to subcategory. **(A)** PFS for subcategories T1a–c. **(B)** PFS for subcategories T1m–e.

The log-rank test revealed a significant difference in PFS (*p* = 0.025; Figure 6A); a significant difference in PFS was observed between T1_c_ and T1_a_ (log-rank adjusted *p* = 0.011) but not between T1_c_ and T1_b_ (log-rank adjusted *p* = 0.415). A significant difference in PFS was observed between T1_e_ and T1_m_ (Figure 6B; Log rank test *p* < 0.001).

**Figure 7:**
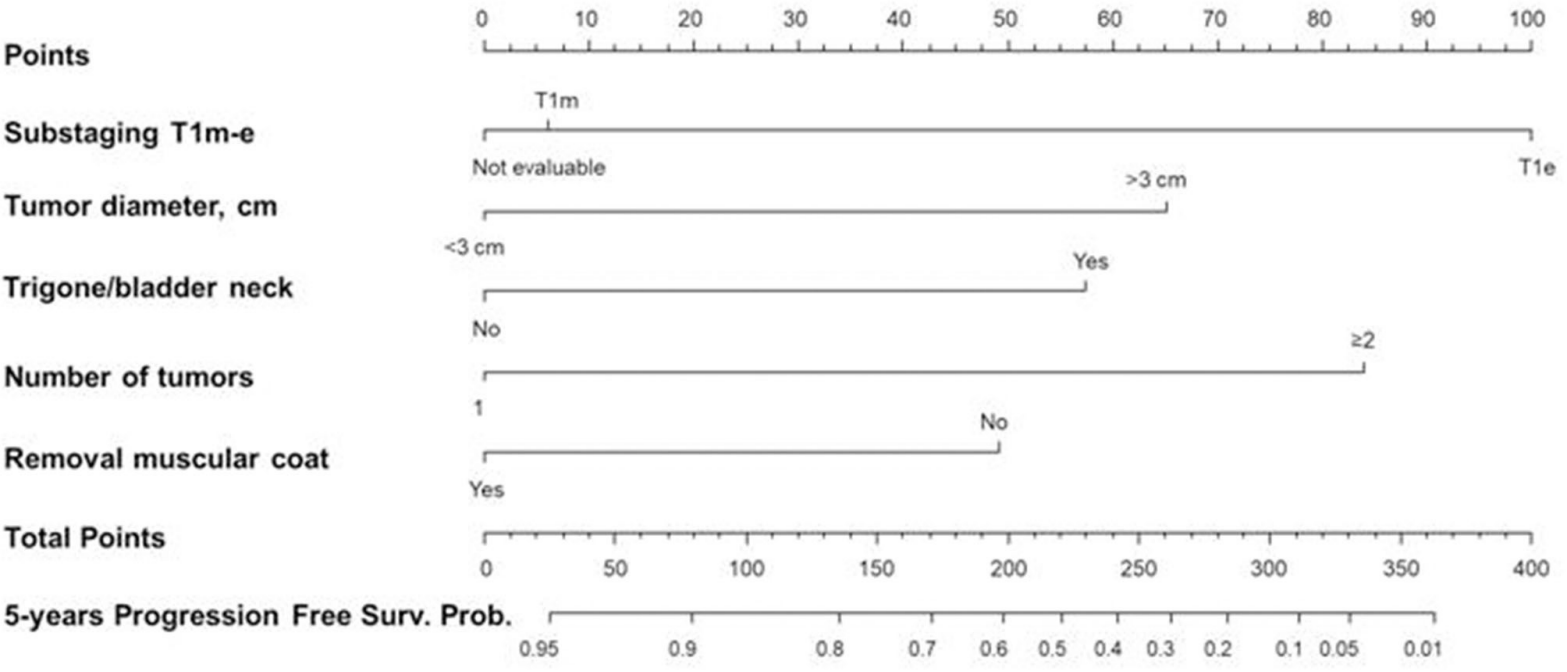
Nomogram 2 for the calculation of the progression-free survival (PFS) at five years. On the basis of the estimated Cox regression model, a patient with T1e (100 points), >3 cm tumor diameter (65 points), trigone/bladder neck location of the tumor (57 points), number of tumors ≥2 (84 points), and with no presence of the muscle layer in the resected specimens (49 points) had a total score of 355 that corresponds to a low 5-year PFS probability (*p* < 0.05).

Harrell's C of M1 was equal to 0.612 and 0.70 for M2. The M2 model showed a slightly higher predictive power than the M1 model (difference = 0.09; *p* = 0.015). Thus, only M2 was considered in the multivariable analysis (Table 7) that was depicted by nomogram 2 (Figure 7).

**Table 7:** Uni and multivariable Cox regression model was performed to evaluate which independent variables influence the progression.

		**Univariable**				Multivariable^*^			
			**95% CI**				**95% CI**		
		**HR**	**LL**	**UL**	**p**	**HR**	**LL**	**UL**	**p**
**Age, yrs**		1	0.97	1.04	0.781				
**Sex**	M vs. F	0.67	0.3	1.51	0.338				
**Smoking**		1.01	0.56	1.83	0.974				
Neutrophil/lymphocyte, *n* = 191		0.89	0.78	1.01	**0.079**	Not in the model			
Trigone/bladder neck		2.66	1.47	4.81	**0.001**	2.04	1.08	3.84	0.028
Tumor diameter, *N* = 203							
	> 3 cm vs. <3 cm	2.45	1.31	4.58	**0.005**	2.35	1.12	4.9	0.023
**N of tumors**								
	≥2 vs. 1	2.98	1.56	5.7	**0.001**	2.89	1.4	5.94	0.004
**Substage T1a-c**									
	T1b vs. T1a	1.48	0.68	3.18	0.32	Not in the model			
	T1c vs. T1a	2.54	1.25	5.15	**0.01**				
	Not evaluable vs. T1a	0.66	0.15	2.88	0.583				
**Substage T1m-e**									
	T1e *vs*. T1m	4.03	2.02	8.03	**<0.001**	3.5	1.56	7.85	0.002
	Not evaluable vs. T1m	0.47	0.1	2.1	0.319	0.69	0.14	3.26	0.636
**Grade**									
	G3 vs. G1	0.71	0.33	1.53	0.377				
CIS		5.24	1.85	14.85	**0.002**	Not in the model			
Muscolaris propria presence		0.53	0.27	1.05	**0.069**	0.52	0.23	1.15	0.107
Lymphovascular invasion, *n* = 203		2.71	1.36	5.38	**0.004**	Not in the model			
Tumor pattern	Solid	3.52	0.48	25.8	0.216				
Squamous metaplasia	Yes vs. no	0.58	0.08	4.2	0.588				
BCG instillations, *n* = 199	Yes	0.73	0.38	1.4	0.34				

*Bold indicate the p < 0.10*.

* *N = 189*.

*All variables with a p < 0.1 (Neutrophil/lymphocyte, Trigone/bladder neck, Tumor diameter, N of tumors, Substage T1m-e, CIS (carcinoma in situ), Lymphovascular invasion, Muscolaris propria presence) were considered for the multivariable analysis; stepwise selection method was applied to obtain the final model*.

**Figure 8:**
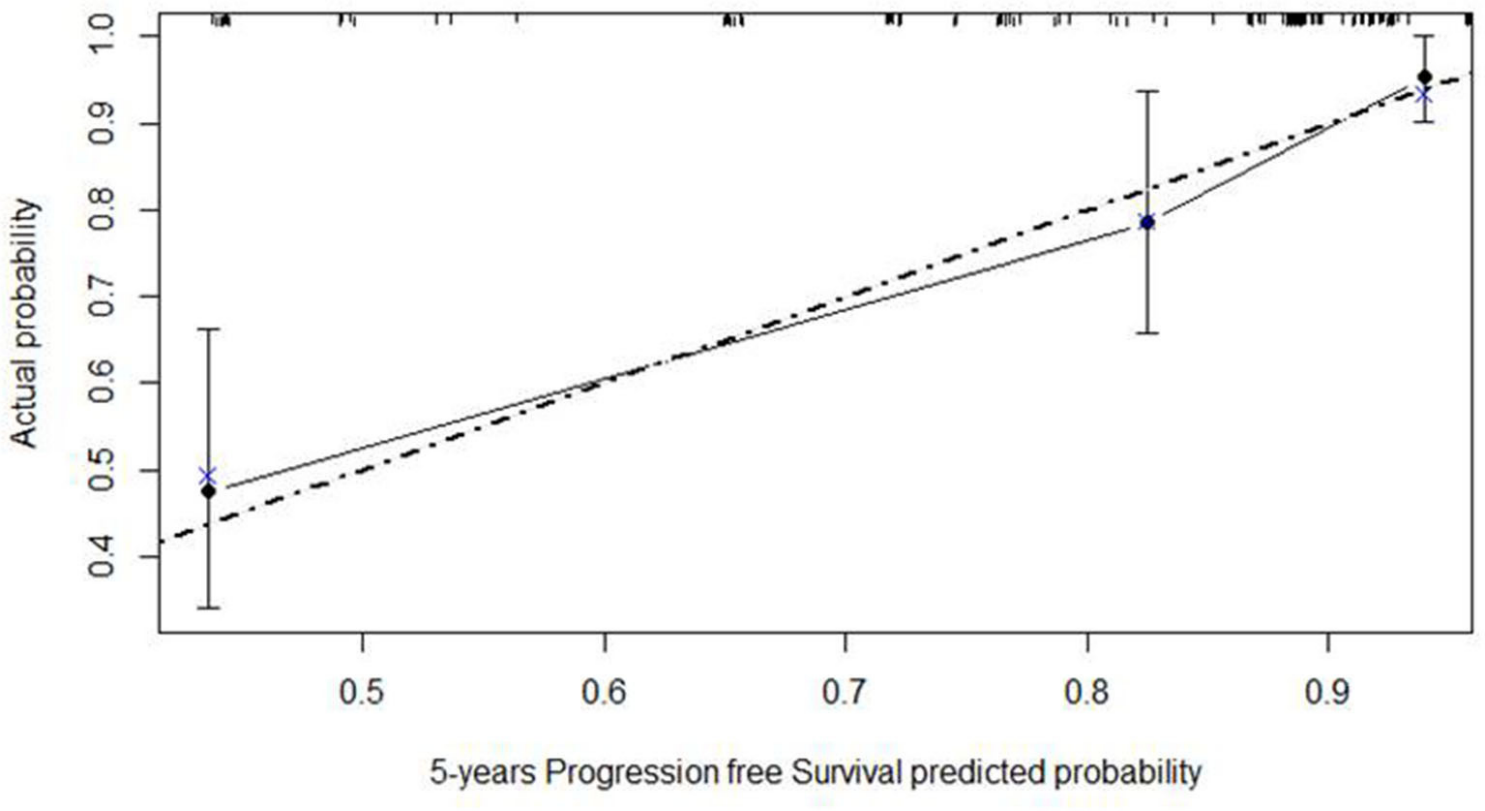
Calibration plot of the Cox regression model of progression-free survival at five years of nomogram 2. The dashed gray line represents the “ideal” line of perfect correspondence between predicted and observed survival, the points represent the predicted “apparent” survival, and the vertical bars represent the 95% confidence intervals. The Xs represent bootstrap estimates corrected with n = 500 resamplings.

The calibration curve is depicted in Figure 8, showing a good agreement between model predictions at 5 years and actual observations.

The BCG showed a significant impact on prolonging PFS only for T1a patients (*p* = 0.032).

## Discussion

The disease T1 BCa is heterogeneous. Although classified as non-muscle-invasive, it is potentially the most aggressive subtype of non-muscle invasive bladder cancer (NMIBC), with high rates of recurrence and considerable rates of progression (4, 5).

The European Organization for Research and Treatment of Cancer (EORTC) risk tables (6) and the Spanish Urological Club for Oncological Treatment (CUETO) scoring model (14) are the two best established predictive tools (“risk calculators”) to help decision-making for patients with NMIBC. However, recent studies that assessed the performance of these predictive tools documented a poor discrimination for both disease recurrence and progression in NMIBC patients, with a tendency of these models to overestimate the risk of disease recurrence and progression in high-risk patients (15). These overestimations remained in BCG-treated patients, especially for the EORTC tables (15).

This overestimated risk may lead to overtreatment with early radical cystectomy for patients with T1 BCa. However, the suboptimal prognostic accuracy of the aforementioned risk calculators may be also associated with the risk of undertreatment, i.e., delays in receiving radical surgery. Consequently, these risk stratification tools may be implemented with new features that could improve their prognostic accuracy and allow for aligning therapy with the real clinical behavior of the individual tumor.

There is growing evidence that tumor depth invasion of the muscolaris mucosae as well as extensive or multifocal invasion of the lamina propria could be such a feature for patients with T1 BCa. A recent meta-analysis evaluated the prognostic value of the subcategorization of oncological outcomes in patients with T1 BCa (8). The prognostic value of pT1 substaging on at least one oncological outcome was established in 29 studies. In seven studies, with a total of 899 patients, MM invasion was associated with a higher disease progression rate (pooled HR of 2.61, 95% CI: 1.61–4.23) (5, 16–21). In six studies, with a total of 930 patients, MM invasion was associated with a higher disease recurrence rate (pooled HR of 1.23, 95% CI: 1.01–1.49) (16–18, 21–23). Tumor infiltration depth was associated with disease progression (pooled HR of 3.29, 95% CI: 2.39–4.51) (2–11, 11–22, 24, 25) and disease recurrence (pooled HR of 1.49, 95% CI: 1.11–2) (21, 22, 25). The meta-analysis concluded that both MM invasion and tumor infiltration depth subcategorization systems were strongly associated with both disease recurrence and progression after adjusting for the effects of established confounding factors (e.g., tumor grade, CIS, and multifocality).

In our study, RFS was significantly lower for T1_c_ (compared with T1_a_) and T1_e_ (compared with T1_m_). The comparison of the two substaging systems for their diagnostic performance in terms of RFS resulted in a slightly higher predictive power for T1_m−*e*_.

The univariate Cox regression model documented that trigone or bladder neck location of the tumor, tumor diameter, number of tumors, substage T1_e_, and squamous metaplasia were significantly associated with recurrence. In the multivariate model subcategory T1_e_, the diameter, location, and number of tumors were confirmed as factors that significantly influenced recurrence after adjusting for the other variables in the model. All these factors are tumor-related and, consequently, not modifiable by the surgeon.

Concerning progression to muscle-invasive or metastatic disease, the log-rank test revealed a significant difference in PFS for T1_c_ (compared with T1_a_) and T1_e_ (compared with T1_m_). The comparison of the two substaging systems, again, yielded a higher prognostic accuracy for the T1_m−*e*_.

The univariate and multivariate analyses confirmed that T1_e_ and the diameter, location, and number of tumors were significantly associated with PFS. The presence of the muscularis propria in the resection specimen (indicating deeper resection) shows a “protective” (pooled HR of 0.52, 95% CI:0.23–1.15), although not statistically significant, effect on tumor progression. Finally, concerning the efficacy of BCG instillations on progression, a protective effect was identified only for T1_a_ (*p* = 0.032).

The nomograms that were tested documented a satisfactory agreement between the model predictions at 5 years and actual observations both for RFS and PFS.

A recent manuscript by Leivo et al. (26) identified five histopathologic features significantly associated with the progression of T1 BCa, irrespective of the presence of muscularis propria: any variant morphology, presence of LVI, necrosis, desmoplasia, and inflammation. Then, the authors used multiple different metrics to quantify T1 invasive tumor burden as either binary (single vs. multiple foci, focal vs. extensive invasion, above MM vs. into vs. below MM) or continuous [the aggregate linear length of invasive cancer (ALLICA), the % of specimen with invasive tumor, the calculated volume of invasive tumor, and the optical micrometer depth]. On the multivariate analysis, only three of these methods remained significant in predicting progression: ALLICA, focal vs. extensive invasion, and relationship to MM. The authors, however, underlined that the use of ALLICA eliminated the influence of the histopathologic features that have been related to progression. Concerning the focal vs. extensive method, the authors underlined the subjectiveness while the MM criteria could not be performed with certainty in 40% of patients.

As a general recommendation, although it is still debatable whether a linear measurement should be used instead of the MM invasion criteria, some strategies of pT1 substaging should still be attempted (13), as the literature clearly demonstrates the correlation between T1 substaging and clinical outcomes.

Finally, in our study, the BCG showed a significant impact on prolonging PFS only for T1_a_ patients. This outcome is in line with the retrospective study of de Jong FC (27); the authors identified 264 patients with high-grade pT1 tumors and subdivided them as having extensive lamina propria invasion (73%) or microinvasion (27%) according to the substaging T1_m−*e*_. With a median follow-up of 68 months, patients with T1_e_ had a statistically significant difference in BCG failure (41 vs. 51%; *P* = 0.002). In the multivariable analysis, T1 sub classification was an independent predictor of high-grade RFS and PFS, indicating that patients bearing tumors with extensive invasion of the lamina propria are more likely to fail BCG therapy. This once again emphasizes the importance of quantifying the invasion of pT1 tumors as information that should be included in every pathology report.

The main limitations of our study are the retrospective design and the low numerosity of the patients combined with a relatively short follow-up, which may have hindered the final analyses and the effect of the BCG on both RFS and PFS for the various subcategories. Moreover, no *en bloc* resection of the BCa was adopted in the current series, which has been proven to improve the identification rate of MM and enhance the accurate identification of the T1 subcategory (28).

The suboptimal adoption of adjuvant BCG instillations is another limitation. In our study, the rate of patients submitted to BCG instillations was 70.4% and, as demonstrated in the univariate analysis (Tables 6, 7), there was not any significant protective effect of the BCG both for progression (HR 0.73, 95% CI: 0.38–1.4, *p* = 0.34) and recurrence (HR 1.4, 95% CI: 0.87–2.25, *p* = 0.162). Although not specifically analyzed since it exceeded the purposes of this manuscript, the reasons for omitting the BCG may have been the higher age-related comorbidities, fear of severe side effects, or even the disbelief of the urologist with respect to the effectiveness of the BCG treatment, mainly for cases treated at the beginning of the current series (years 2009–2012). The suboptimal adoption of BCG instillations, however, has been commonly reported in the published literature. A recent population-based Scandinavian study (29) reports the total rate of T1 patients treated with BCG either early or delayed as 41%, with only 15% receiving BCG as early treatment (within 8 weeks after final diagnosis). In a Swedish T1 nationwide population-based study, the BCG rate was somewhat higher at ~50% (30).

Low rates of re-TUR within 4 weeks and the absence of routine mapping during the repeated resection of the bladder are other limits of the study. With a strict definition of re-TUR as performed within 2–6 weeks from the first resection, only 20.2% of patients were submitted to re-resection. The reasons for the low uptake of re-TUR may be different; it may have been avoided, especially for low-grade disease at the initial TUR (the rate of G1 in this series was 12%) in elder or medically complex patients with severe comorbid conditions; other reasons for TUR avoidance may have been patient refusal or simply logistical reasons that prevented a re-TUR within the 8 week threshold. However, 167 (81.9%) of our pathologic examinations reported the presence of the muscularis propria in the resection specimen (with a consequent reduced risk of disease understaging), which is a significantly higher number with respect to other studies where the rate is <40% at the initial resection (31). In the latter study, the reported rate of RE-TUR was 22.4%. Low rates of re-TUR are commonly reported in published literature, reflecting the difficulties of adopting current guidelines in real-life practice (32).

The development of new risk-calculators that incorporate both the T1 subcategorization and the T1 molecular subtype as described by Robertson et al. (33), preferably on *en bloc* resection specimens, may overcome the limitations of the currently used models that show a poor discrimination for both disease recurrence and progression in NMIBC patients.

## Conclusions

Both subcategorization systems (T1_a−c_ and T1_m−e_) are significantly associated with disease progression and recurrence for patients affected by T1 BCa, with T1_m−*e*_ showing a slightly higher prognostic performance. Adequately designed prospective studies are necessary for the development of innovative risk calculators for the prediction of disease recurrence and progression and risk of BCG failure in T1 BCa; these models should incorporate, besides the “traditional” prognostic factors, the T1 substaging.

## Data Availability Statement

The raw data supporting the conclusions of this article will be made available by the authors, without undue reservation.

## Ethics Statement

The studies involving human participants were reviewed and approved by Policlinico Tor Vergata ethics board, registration number 255.19. Written informed consent for participation was not required for this study in accordance with the national legislation and the institutional requirements.

## Author Contributions

AA provided substantial contribution to the conception and design of the work as well as analysis and interpretation of data and drafting of the manuscript. AA, GCo, RT, GCa, AF, AM, RM, SD, SG, EF, VP, AC, and AP have revised the work critically for important intellectual content and provided final approval of the version to be published. They agree to be accountable for all aspects of the work in ensuring that questions related to the accuracy or integrity of any part of the work are appropriately investigated and resolved. GCo, RT, GCa, and AF provided substantial contributions to the acquisition and analysis of data for the work. All authors contributed to the article and approved the submitted version.

## Conflict of Interest

The authors declare that the research was conducted in the absence of any commercial or financial relationships that could be construed as a potential conflict of interest.

## Publisher's Note

All claims expressed in this article are solely those of the authors and do not necessarily represent those of their affiliated organizations, or those of the publisher, the editors and the reviewers. Any product that may be evaluated in this article, or claim that may be made by its manufacturer, is not guaranteed or endorsed by the publisher.
